# Isolation and characterization of native *Bacillus thuringiensis* strains from Saudi Arabia with enhanced larvicidal toxicity against the mosquito vector *Anopheles gambiae* (*s.l.*)

**DOI:** 10.1186/s13071-016-1922-6

**Published:** 2016-12-19

**Authors:** Talaat A. El-kersh, Ashraf M. Ahmed, Yazeed A. Al-sheikh, Frédéric Tripet, Mohamed S. Ibrahim, Ali A. M. Metwalli

**Affiliations:** 1 Department of Clinical Laboratory Sciences, College of Applied Medical Sciences, King Saud University, Riyadh, Saudi Arabia; 2 Department of Zoology, College of Science, King Saud University, Riyadh, Saudi Arabia; 3 Centre for Applied Entomology and Parasitology, School of Life Sciences, Keele University, Staffordshire, ST5 5BG UK; 4 Department of Food Science & Nutrition, College of Food and Agricultural Sciences, King Saud University, Riyadh, Saudi Arabia

**Keywords:** *Bacillus thuringiensis*, Isolation, Parasporal crystals, Biochemical type, 16S rRNA, *Anopheles gambiae*, *cry* and *cyt* genes, SDS-PAGE

## Abstract

**Background:**

Worldwide, mosquito vectors are transmitting several etiological agents of important human diseases, including malaria, causing millions of deaths every year. In Saudi Arabia, as elsewhere, vector-control is based mostly on chemical insecticides which may be toxic and cause environmental deprivation. Here, to support the development of bio-pesticide alternatives, a study was conducted to identify native *Bacillus thuringiensis* (*Bt*) isolates with improved toxicity against the malaria vector, *Anopheles gambiae* (*s.l*.).

**Methods:**

Sixty-eight *Bt* isolates were obtained from 300 soil and other samples collected from 16 sites across Saudi Arabia. *Bt* identification was based on morphological characteristics of colonies, shape of parasporal crystals and biochemical profiles. After characterization of their mosquitocidal activity, larvicidal strains were described through 16S ribosomal DNA gene sequencing, *cry*, *cyt* and *chi* genes PCR-amplification profiles, and SDS-PAGE protein analyses.

**Results:**

Spherical *Bt* crystals were predominant amongst the 68 isolates (34%), while irregular, bi-pyramidal and spore-attached crystals were found in 32, 13 and 21% of strains, respectively. LC_50_ and LC_90_ bioassays showed that 23/68 isolates were larvicidal, with distinct biochemical activity profiles compared to non-larvicidal *Bt* strains. Eight larvicidal strains showed larvicidal activity up to 3.4-fold higher (LC_50_ range: 3.90–7.40 μg/ml) than the reference *Bti-*H14 strain (LC_50_ = 13.33 μg/ml). Of these, 6 strains had *cry* and *cyt* gene profiles similar to *Bti*-H14 (*cry4Aa, cry4Ba, cry10, cry11, cyt1Aa, cyt1Ab, cyt2Aa*). The seventh strain (*Bt*63) displaying the highest larvicidal activity (LC_50_ = 3.90 μg/ml) missed the *cry*4Aa and *cyt*1Ab genes and had SDS-PAGE protein profiles and spore/crystal sizes distinct from *Bti-*H14. The eight strain (*Bt*55) with LC_50_ of 4.11μg/ml had *cry* and *cyt* gene profiles similar to *Bti*-H14 but gave a *chi* gene PCR product size of 2027bp. No strains harbouring *cry2, cry17 + 27, cry24 + 40, cry25, cry29, cry30,* or *cyt2Ba* were detected.

**Conclusion:**

This study represents the first report of several Saudi indigenous *Bt* strains with significantly higher larvicidal efficacy against *An. gambiae* than the reference *Bti*-H14 strain. The very high toxicity of the *Bt*63 strain, combined with distinct *cry* and *cyt* genes and SDS-PAGE-protein profiles makes it a promising candidate for future applications in mosquito bio-control.

**Electronic supplementary material:**

The online version of this article (doi:10.1186/s13071-016-1922-6) contains supplementary material, which is available to authorized users.

## Background

Mosquitoes are considered as one of the most important groups of insects that are transmitting the parasites and viruses responsible for many major infectious diseases such as malaria, filariasis, dengue, Rift Valley and yellow fevers, and Japanese encephalitis, which contribute significantly to poverty and social debility in as much as 128 tropical countries (55% of the world’s population) causing millions of deaths every year. In the absence of effective vaccines or specific anti-viral drugs for the majority of these diseases, the effective alleviation of this burden has often crucially relied on mosquito control programs [1]. The extensive and intensive use of chemical insecticides for mosquito control has been the cause of several environmental and human health concerns, including disruption of natural-biological control systems, development of mosquito resistance and undesirable effects on beneficial organisms [2]. The awareness of these problems has led developed countries to implement rigorous control on the use of harmful chemical control measures almost 10 years ago [1, 3]; however, in the Middle East and developing countries the same measures have not always been implemented.

One of the few alternatives to chemical compounds for vector control is *Bacillus thuringiensis* (*Bt*). This facultative aerobic, Gram-positive, spore-forming saprophyte soil bacterium, has been successfully used as a biological insecticide over the last 6 decades and constitutes 95% of all commercial bio-insecticides, due to its high specificity, safety and effectiveness in the control of wide spectrum of human disease vectors and agriculture-pests [4]. Spore-formation enables *Bt* to survive in harsh environments resulting in a ubiquitous distribution. *Bt* has been isolated from soil, aquatic environments including sewage, dead insects and their breeding sites, herbivore faeces, stored grains, phylloplane and forest [4]. Its persistence is thought to be comparatively shorter than that of the closely-related bacterium *Lysinibacillus sphaericus.* During sporulation, *Bt* produces one or more parasporal insecticidal proteinaceous crystals (ICPs), comprised of one or more crystal (*Cry*) and cytolytic (Cyt) proteins, recognized as δ-endotoxins. In target insect species, ingestion of ICPs, is followed by their dissolution under prevailing alkaline pH in the larvae midgut and release of protoxins in the lumen that are activated by specific proteases to recognize and bind to receptors from the midgut microvilli. After binding, the toxin molecules go through oligomerization and membrane insertion that cause pore formation, and lead to eventual cell-cytolysis [5–7]. The formation of ICPs is the only phenotypic character that differentiates the two taxonomically closely related species, *Bt* and *Bacillus cereus* [8, 9], which have been proposed to be considered as a single species [10]. Hofte & Whitely [11] were the first to describe four Cry and two Cyt δ-endotoxins genes on the basis of insecticidal activity. Since genetic diversity and toxic potential of *Bt* strains has been shown to vary between countries and regions, hundreds of *Bt* strains have been isolated and characterized all over the world, with the aim to find novel active *cry* genes to combat the emergence of resistant insects, primarily among Lepidoptera. Nowadays, *Bt* Cry toxins have been classified into 73 families (Cry1 to Cry73) and six groups of Cry proteins with toxicity to various insect taxa. Cyt proteins have been classified into three families (Cyt1, Cyt2 and Cyt3) with specific toxicity against mosquitoes and blackflies [12, 13]. The latter cytotoxins are hydrophobic, show no homology to Cry proteins, and, even at low concentration, enable Cry toxins to create oligomeric pores in the cell membranes of the insect gut. Therefore, they synergize with and overcome resistance to mosquito larvicidal Cry proteins by functioning as a Cry membrane bound receptor. At high concentrations, due to their high affinity to lipids, these toxins can act as detergents by rupturing the cell membrane [6, 7, 14]. Additionally, the chitinase *chi* gene has been associated with enhanced mosquitocidal activity of *Bt* strains suggesting further synergism with the *cyt* and *cry* genes [7].

*Bacillus thuringiensis* serovar*. israelensis* de Barjac (*Bti-*H14) was the first *Bt* strain used as an effective biological control agent against larvae of many mosquito and blackfly species worldwide, with no adverse effects on non-target invertebrates and vertebrates. Several studies have linked the larvicidal activity of *Bti* to its major Cyt1Aa and Cry4Ba, Cry4Aa, Cry10, Cry11Aa proteins [1, 15, 16]. In *Bti*-H14, these proteins are all encoded and located on a large plasmid (128 kb) recognized as p*Bt*oxis [17] and differ in their mosquito toxicity [18, 19]. All four *Cry*-toxins, Cry4A, Cry4B, Cry10 and Cry11A are synthesized at different phases of sporulation and are added sequentially to form spherical ovoid inclusion bodies [4]. The Cry4A and Cry4B toxins have large molecular masses of (128 to 134 kDa), hence easily form crystals. However, Cry10 and Cry11 have lower mass of 78 and 72 kDa (naturally truncated) and require the helper proteins, encoded by genes (*p*19 and *p*20) in the same *cry* operon, to facilitate their crystallization [17, 20]. Both Cry4A and Cry4B, after dissolution in the alkali mosquito-larval midguts, are proteolytically cleaved into a C-terminal half and N-terminal half. Only the N-terminal halves are toxic and can be genetically manipulated to broaden such toxicity [21], whereas the C-terminal halves crystallization domains are not involved in toxicity but are necessary for crystal formation, as strategy for protection against highly intrinsic proteolytic enzymes produced concomitantly by the producer organism [20]. The larvicidal toxicity of individual Cry proteins is low and does not reach more than 5% that of the complete parasporal body, thereby highlighting their synergetic effect [18, 22].

Despite having been used for mosquito control for three decades, the risk of emergence of resistance to *Bti* is thought to be limited due to its unique combination of Cry toxin crystals combined with Cyt toxins, which leads to complex multiple mode of actions and multiple levels of synergistic interactions. However, that risk is not completely nil and recent reports have indicated decreased susceptibilities in some field populations suggesting that future wider use of *Bti* might cause the emergence of mosquito-resistance [18, 19, 23].

Although the majority of natural *Bt* isolates with insecticidal activity are effective against lepidopteran larvae, strains active against dipterans are comparatively more difficult to obtain [24]. In a recent report, we succeeded in isolating native *Bt* lines from Saudi Arabia with significantly enhanced activity against *Aedes caspius* and slightly enhanced activity against *Culex pipiens* [25]. Therefore, as a continuation to our interest in the control of mosquito-borne diseases in Saudi Arabia and elsewhere [24, 25], this research effort was expanded to identify and characterize additional indigenous *Bt* isolates with naturally enhanced toxicity for bio-control of the malaria mosquito *An. gambiae*. To the best of our knowledge, this is the first report of native *Bt* strains native to Saudi Arabia with significantly enhanced larvicidal activity against an anopheline mosquito compared to the reference *Bti-*H14 strain [1, 24] and with distinct SDS-PAGE and *cry* and *cyt* gene profiles. It is hoped that the findings of the present study may contribute to the Saudi Ministry of Health’s efforts for the control of mosquito-borne diseases, and may be useful to vector control programmes in other parts of the world, where these mosquito vectors are prevailing.

## Methods

### Field collection of environmental samples

From October 2013 to March 2014, environmental samples (*n* = 300) were collected in sterile plastic universal tubes from 16 regions across Saudi Arabia in locations in the vicinity of mosquito breeding sites (Table 1). Samples were collected near houses, irrigated parks, gardens and farms as well as from the surrounding semi-desert areas. Collected samples were transported to the laboratory and stored at 4 °C until processed for *Bt* isolation.

**Table 1:** Geographical distribution of sampling sites, total number of samples (*N*), and number of *Bt*-positive, *Bt* index, and number of larvicidal isolates

City-region	Geographic location (GPS)	*N*	*Bt*-positive	*Bt* index^a^	Larvicidal
Al Madinah	24°28′0″N, 39°36′0″E	52	18	0.43	12
Yanbu	24°5′0″N, 38°0′0″E	32	5	0.12	2
Makkah	21°30′0″N, 41°0′0″E	22	7	0.24	2
Taif	21°16′0″ N, 40°25′0″E	14	5	0.66	0
Jeddah	21°50′0″N, 39°10′0″W	2	0	–	0
Qassim	25°49′20″N, 42°50′7″E	39	4	0.23	1
Alehssa	25°25′46″N, 49°37′19″E	9	2	0.67	0
Dammam	26°26′0″N 50°07′0″E	7	0	–	0
Qatif	26°56′0″N, 50°1′0″E	6	0	–	0
Hafr al Batin	28°26′3″N, 45°57′49″E	22	4	0.21	1
Riyadh region	24°38′0″N, 46°43′0″E	37	9	0.22	1
Asser	19°0′0″N, 43°0′0″E	13	4	0.15	2
Khamis Musheet	18°18′0″N, 42°44′0″E	8	1	0.68	0
Abhaa	18°13′24″N, 42°30′26″E	10	3	0.13	0
Jezan	16°53′21″N, 42°33′40″E	25	5	0.70	2
Najran	17°29′30″N, 44°7′56″E	2	1	0.12	0
Total		300	68	0.35	23

^a^The number of identified *Bt* colonies divided by the total number of spore-forming colonies

### Bacterial isolation and culturing

Around 1 g of fine grinded soil, dried leaves or animal-faeces, was added to 2.0 ml of sterile distilled water and suspended vigorously using a Vortex Mixer; whereas, 2 ml of liquid samples such as, for example, sewage water were transferred to sterile tubes. Of these specimens, 2 ml aliquots were mixed with 2 ml absolute ethanol to obtain 50% ethanol concentration, vortexed for 1 min and then incubated at 30 °C for 45 min with regular shaking. After ethanol treatment, 1/10 serial dilutions in sterile distilled water were carried out (1 × 10^-1^–10^-3^) and spread on synthetic nutrient agar (SNA) medium supplemented with 0.2% yeast extract (Sisco research laboratories, Mumbai, India) and 0.005% of manganese chloride (MnCl_2_), and incubated at 30 °C for 2 to 3 days depending on spore maturation [24, 25].

### *Bt* colony morphology analysis

*Bt*-like colonies, white, large, nearly circular with fine irregular margins and may be glossy, less glossy or rough were selected. To exclude closely related *B. cereus* and other spore-forming bacilli, the suspected colonies were then examined under phase contrast microscopy for the presence of parasporal crystals. For comparison, the *Bt* index was calculated for each positive sample as number of identified *Bt* colonies divided by the total number of spore-forming bacilli colonies [24, 25].

### Biochemical activity typing

In addition to spore formation, positive Gram staining, and parasporal crystal detection, further phenotypic characterization and identity confirmation of recovered 68 *Bt* isolates were accomplished on the basis of esculin and lecithinase hydrolysis, hemolytic and motility activities, and carbohydrate utilization (API 50CH system) as previously described [24]. The resulting API data analysis confirmed the identity (95–99%) of native *Bt*-like isolates as *Bacillus thuringiensis* strains*.*

### *Bt* crystal morphology analysis

Next, confirmed *Bt* colonies were suspended in sterile water, wet mounted, and then examined under Phase Contrast Microscope at a 100× magnification for presence of parasporal crystals. They were then purified by sub-culturing on SNA medium for 48 h and stored at -20 °C as stock culture in sterile liquid nutrient broth containing 50% glycerol. Subsequent morphometric measurements of spore or crystal sizes were performed with Scanning Electron Microscopy (SEM) as previously described [24].

### Mosquito maintenance

*Anopheles gambiae* (*s.l*.) mosquitoes were maintained at the Centre for Applied Entomology and Parasitology at Keele University, United Kingdom under standardized conditions as detailed in [26]. Adult female mosquitoes were fed on horse blood using an artificial feeder (Hemotek membrane feeding system, Discovery workshops, UK) set at 37 °C [26]. Eggs were laid 2 days after blood-feeding and hatched within 2 days. About 200 hatched first larval instars were distributed into 1L of de-ionized water in plastic trays (34 × 24 cm each). One drop of Liquifry (Interpet Ltd, Dorking, UK) was added to each dish for 2 days then, were fed on ground TetraMin flake food (Tetra Werke, Melle, Germany) until pupation. Pupae were separated daily, placed in polystyrene pots containing distilled water, and kept in their rearing cages until adult emergence [26].

### *Bt* spores/crystals mixture and preliminary mosquito-bioassay

The spores and crystals mixture of each native *Bt* isolates cultured on SNA plates (72 h) were scrapped into 10 ml of sterile water, to give an average count of 10^9^ colony forming units (CFU) per ml. At the beginning a high spores/crystals mixture was used in parallel with that of the *Bt* serovar *israelensis* de Barjac reference strain (*Bti-H14*) [24] in preliminary screening of larvicidal activity.

For preliminary bioassays, groups of 20 third-instar larvae were transferred into 30 ml plastic cups containing 20 ml of chlorine-free tap water using a Pasteur pipette. To detect even low larvicidal activity, the crude *Bt* spores/crystals mixture (~1 × 10^8-9^ CFU/ml) was then added and larval mortality of tested isolate was scored 24 h after incubation at 22 ± 1 °C and compared to that of the negative control. Out of the tested native *Bt* isolates, those that exhibiting significant mosquito-larvicidal activity (100% mortality), were selected for further bioassay assessment and spore counts.

### 16S rRNA gene analysis

Fresh colonies of a sample of 23 isolates (19 larvicidal, 4 non-larvicidal), as well as *B. cereus* (ATCC1177) and the reference *Bti*-H14 strains, were picked up with a sterilized toothpick, and suspended in 0.5 ml of sterilized saline in a 1.5 ml centrifuge tube, centrifuged at 3,500× *g* for 10 min. After removal of supernatant, the pellet was suspended in 0.5 ml of InstaGene Matrix (Bio-Rad, USA), incubated at 56 °C for 30 min and then heated at 100 °C for 10 min. After heating, supernatant DNA was used for PCR 16S rRNA gene analysis. PCR amplification of 16S rRNA gene from tested *Bt* isolates was performed using the universal primers: forward (518F): 5'-CCA GCA GCC GCG GTA ATA CG-3'; reverse (800R): 5'-TAC CAG GGT ATC TAA TCC-3', essentially as previously described [24]. The purified PCR products of approximately 1,400 bp were sequenced using Big Dye terminator cycle sequencing (Macrogen, Seoul, Korea) with 2 primers (27F): 5'-AGA GTT TGA TCM TGG CTC AG-3' and (1492R): 5'-TAC GGY TAC CTT GTT ACG ACT T-3'). Sequences were searched against the NCBI Nucleotide database (www.ncbi.nlm.nih.gov) using the BLAST tool with a similarity cut-off of 99.5% to identify the bacterium based on sequence homology. Using the programme Clustal-X [27], a multiple alignment was build using the 16S RNA gene sequences of larvicidal and non-lethal *Bt* isolates, the sequences from the colony-grown *Bti*-H14 reference and *B. cereus* strains, and sequences from the genomes of *B. pumilus*, and *B. megatorium*, used as Gram-positive bacteria outgroups (Additional file 1: Table S1) [24]. The software Seaview4 [28] was used to build a boot-strapped neighbour-joining tree in order to establish the relative degree of genetic similarity between the sequences.

### Detailed mosquito larvicidal bioassays

To avoid possible discrepancy during the preparation of spores/crystals mixture for quantitative determination of LC_50_ and LC_95_ mortality bioassay, the spores/crystals mixtures of the active 23 native *Bt* isolates were prepared from fermentation growth on Nutrient Yeast Extract Salt Medium, NYSM (containing per litter: 5 g glucose, 5g peptone, 5 g NaCl; 3 g beef extract, 0.5 g yeast extract, 0.02 g magnesium chloride, 1 mg manganese chloride and 0.01 g calcium chloride, pH 7.2) and prepared as dried powder in adequate amount using the lactose acetone co-precipitation procedure [29]. The resulting white fine toxin powders were stored at 4 °C.

For detailed characterization of mosquito larvicidal activity, the LC_50_ and LC_95_, 24 h post-treatment of the native larvicidal *Bt* isolates and *Bti*-H14 reference strain were established using bioassays conducted on third-instar larvae of *An. gambiae*. For each bioassay, five concentrations from each active toxin powder were used as recommended by WHO [35] with modifications. Briefly, 20 third-instar larvae were placed in each well of a sterile standard 12-wells tissue culture test plate (Nunclone Delta Surface, Thermo-Fischer Scientific, Denmark) with 2 ml de-ionized water mixed with either 10 μl of each dilution of toxin powder solution [36] or 10 μl de-ionized water for the negative control larval group. Another set of larvae groups were treated with the reference *Bti-*H14 strain as positive control for comparison. Meanwhile, samples from each of the same serial dilutions of *Bt* toxin were cultured on SNA medium to estimate spore counts by counting the number of CFU/μg. Each bioassay was run for 24 h, during which larvae were fed to avoid mortality caused by starvation. After 24 h, the percentage of larval mortality was calculated for each concentration using Abbott’s formula [37]. Bioassays comprising one replicate of each concentration were replicated five times for every strain tested.

### DNA extraction for PCR detection of *cry* and *cyt* genes contents

A single fresh colony of each larvicidal *Bt* isolates and the reference *Bti*-H14 [24], were suspended into 100 μl sterile distilled water in an Eppendorf tube and boiled in water-bath for 10 min. *Bt* suspensions were then immediately cool-shocked at -20 °C. This heat-shock process was repeated three times to allow complete cell-lysis before centrifugation (Multifuge 3SR+ Centrifuge, Thermo Scientific Electron Corporation, Germany) at 3,500× *g* for 10 min at 4 °C. The resultant supernatant containing the crude DNA was used for PCR amplification, using the Hot Start Taq DNA polymerase and Master Mix Kit (QIAGEN Technologies, Germany) as described in Bukhari & Shakoori [30].

Each native *Bt* isolates and the reference *Bti*-H14 strains were PCR-screened using five universal primers designed from conservative regions of the *cry2*, *cry4*, *cry11*, *cyt1* and *cyt2* genes as previously described [31–33] as well as 13 primers specific to highly variable regions of the *cry4a*, *cry4ba*, *cry10*, *cyt1Aa*, *cyt1Ab*, *cyt2Aa*, *cyt2Ba*, *cry17* + *27*, *cry24* + *40*, *cry25*, *cry29*, *cry30* (Additional file 2: Table S2). The *chi* gene, known for its chitinase activity that may increase insecticidal toxicity [7], was also examined using a set of specific primers (Additional file 2: Table S2)*.* Oligonucleotide primers were synthesized in a DNA synthesizer (Applied Biosystems, Foster City, CA, USA) using the manufacturer’s instructions. PCR amplifications were performed as previously described [25] and the resulting amplicons were visualized by running the PCR products (15μl) on 2% agarose electrophoretic gels as described in Mahalakshmi et al. [34].

### SDS-PAGE analysis

The SDS-PAGE protein profiles of *Bt* isolates and the references *Bt*-H14 were established as previously described in Bukhari & Shakoori [30] and El-Kersh et al*.* [25].

### Data analysis

All statistical analyses for calculating LC_50_, LC_95_, slopes, and standard error values of each treatment were undertaken according to Finney [38]. Any two relevant treatments were considered as not significantly different in their toxicity if the 95% confidence intervals of their LC_50_ overlapped [39].

## Results

### Geographical distribution of positive *Bt* isolates

A total of 300 samples were collected from 16 different regions of the Kingdom of Saudi Arabia (Table 1) and screened for *Bt* presence. A total of 68 native *Bt* isolates were recovered from 55 samples of soil, dried plant leaves, dried animal dung, sewage water, irrigation water, stagnant rain water, insects, snails and fish guts. Another 245 samples failed to yield any *Bt* isolates. Of the 55 *Bt*-positive samples, some contained several *Bt* strains which diverse colonial morphology and parasporal crystal shapes, whereas other samples only a single *Bt* strain. The highest diversity of *Bt* isolates were recovered from Madinah, Jezan, Asser and Yombu regions. Other city/regions yielded positive *Bt* isolates but with comparatively lower diversity regardless of respective *Bt* indices (Table 1).

### Morphological characterization of *Bt* colonies

Wet mount phase-contrast microscopy allowed the identification of 68 native potential *Bt* isolates with characteristic *Bt*-like colonial morphology and presence of parasporal crystals. Of these, only 23 exhibited larvicidal activity in preliminary bioassays. The colony growth of these strains on SNA medium bore strong similarities with those of the reference *Bti-*H14, showing white colour, large size, nearly circular shape with fine irregular margins and usually glossy. In contrast, the 45 non-larvicidal *Bt* isolates showed much greater morphological variation. However, all native *Bt* isolates shared many morphological characteristics in cell chains arrangement, ellipsoidal shape of spores, and non-swollen sporangia. As expected, both the 23 larvicidal and 45 non-larvicidal native *Bt* isolates exhibited remarkable variation in the position and shape of crystals within sporangia. Based on this variation, the 68 native *Bt* isolates were broadly classified into four classes: (i) small and/or large spherical (34%); (ii) bi-pyramidal (13%); (iii) irregular: cubic, spherical, merged triangular, or conical-budding (32%); and (iv) spherical, triangular, cubic or bi-pyramidal attached to spores (21%).

### Biochemical typing

In a previous study, biochemical typing of 3,639 *Bt* isolates [40] showed that urease producers were strongly associated with the production of lepidopteran-toxic bipyramidal crystals, whereas amorphous and/or irregular crystal formers with dipteran toxicity, were mostly associated with either general low metabolic activity, positive for acid production from starch and lecithinase and/or esculine hydrolysis. Here, the 68 native *Bt* isolates were negative for H_2_S, urease, and indole production and rarely positive for ONPG or acid production from rhamnose (Table 2). *Bt* isolates showed great variation in their ability to utilize citrate (25%), to reduce nitrate (62%) and produce acid from starch (75%), sucrose (65%), mannitol (45%), inositol (44%) and sorbitol (39%). Most isolates were positive for gelatinase liquification, esculin hydrolysis and lecithinase production, hemolytic (94%) and motile (90%) activities. However, all 23 larvicidal *Bt* isolates (100%) exhibited active motility and hemolytic activity as well as lecithinase and esculin hydrolysis, but were negative for salicin.

**Table 2:** Biochemical profiles of the 68 native *Bt* isolates from Saudi Arabia using the API 50CH system. The percentage of isolates actively metabolizing different substrates is indicated

100% positive	> 90% positive	10–90% positive	< 10% positive	100% negative
L-Tryptophane	L-Arginine (99)	Citrate (25)	Rhamnose (4)	L-Lysine
Gelatin	Glucose (95)	Sodium pyruvate (69)	ONPG (1)	L-Ornithine
Esculin		Mannitol (45)		Sodium thiosulfate
		Inositol (44)		Urea
		Sucrose (65)		Indole
		Sorbitol (39)		Salicin
		Starch (75)		Melibiose
		Nitrate (62)		Arabinose

### Larvicidal bioassays

Preliminary screening of larvicidal bioassays revealed that only 23/68 (34%) of *Bt* isolates showed promising mosquito larvicidal activities and these were further characterized with regard to their toxicity. The results of detailed LC_50_ and LC_95_ bioassays and spore count (CFU per μg powder) confirmed that all 23 native *Bt* isolates identified as larvicidal in preliminary test had various level of efficacy against *An. gambiae* third-instar larvae (Table 4). The LC_50_ of most (*n* = 15) of the tested 23 *Bt* isolates did not significantly differ than that of the reference *Bti-*H14 (LC_50_: 13.33 μg/ml and spore count of 4.5 ± 0.3 × 10^5^ CFU/μg powder). Notably, as much as eight native *Bt* strains exhibited significantly higher larvicidal activity against *An. gambiae* than that of *Bti*-H14 reference strain. These isolates coded *Bt* No. 63, 55, 10, 68, 58, 42, 67 and 57, showed LC_50_ of 3.90, 4.11, 4.96, 5.16, 5.73, 5.90, 6.90 and 7.40 μg/ml, respectively (Table 3). Their respective LC_95_, higher Probit regression slope values and their CFU-spore counts per μg of powder similar or lower than the reference *Bti-*H14 strain confirmed their higher toxicity.

**Table 3:** Toxicity of 23 *Bt* isolates and the *Bti-*H14 reference strain against third-instar larval stage of *An. gambiae* 24 h post-treatment

*Bt*-code	Region	LC_50_ (μg/ml) ^a^	LC_95_ (μg/ml) ^a^	Slope ± SE	CFU/μg × 10^5 b^
*Bti*-H14	–	13.3 (11.2–15.9)	63.7 (40.4–102.3)	2.4 ± 0.07	4.5 ± 0.3
*Bt*05	Kharj	13.4 (11.07–16.5)	78.5 (46.0–137.0)	2.15 ± 0.06	1.0 ± 0.1
*Bt*07	Asseer	15.0 (12.2–18.8)	90.4 (50.5–166.0)	2.12 ± 0.07	1.0 ± 0.2
*Bt*10	Qassim	5.0 (4.4–5.6)^*^	18.6 (14.6–23.8)	2.90 ± 0.06	1.2 ± 0.3
*Bt*11	Yanbu	12.3 (10.2–14.8)^*^	72.6 (43.5–124.3)	2.10 ± 0.06	0.65 ± 0.3
*Bt*12	Yanbu	9.5 (8.3–10.9)^*^	41.6 (29.4–59.6)	2.60 ± 0.06	0.53 ± 0.4
*Bt*16	Asser	13.0 (10.6–16.0)	84.2 (47.8–152.6)	2.02 ± 0.06	0.92 ± 0.3
*Bt*17	Hafr-elbaten	22.0 (18.2–26.7)	128.5 (76.4–219.7)	2.14 ± 0.06	8.1 ± 0.4
*Bt*26	Madinah	12.9 (9.9–16.9)	149.7 (64.7–367.2)	1.54 ± 0.05	4.1 ± 0.7
*Bt*27	Madinah	7.1 (6.1–8.2)^*^	40.1 (27.3–60.2)	2.20 ± 0.05	5.9 ± 0.4
*Bt*28	Makkah	8.6 (7.7–9.6)^*^	27.5 (22.4–33.9)	3.30 ± 0.07	6.2 ± 0.3
*Bt*29	Madinah	14.4 (12.4–16.7)	49.4 (34.1–72.3)	3.10 ± 0.12	0.52 ± 0.1
*Bt*42	Jezan	5.9 (5.2–6.8)^*^	24.3 (18.9–31.3)	2.70 ± 0.07	0.93 ± 0.3
*Bt*44	Jezan	16.1 (13.7–18.9)	91.9 (59.3–144.2)	2.20 ± 0.05	0.63 ± 0.4
*Bt*53	Madinah	13.8 (11.7–16.4)	101.7 (61.3–171.9)	1.90 ± 0.05	1.3 ± 0.4
*Bt*55	Madinah	4.1 (3.6–4.6)^*^	14.4 (11.6–18.1)	3.02 ± 0.07	1.95 ± 0.3
*Bt*56	Madinah	11.7 (10.2–13.5)^*^	43.8 (31.2–62.2)	2.90 ± 0.08	0.15 ± 0.3
*Bt*57	Madinah	7.4 (6.5–8.25)^*^	26.1 (20.9–32.9)	2.98 ± 0.07	1.27 ± 0.3
*Bt*58	Madinah	5.7 (5.1–6.5)^*^	24.6 (18.6–32.9)	2.60 ± 0.05	1.24 ± 0.2
*Bt*59	Madinah	10.3 (8.5–12.6)^*^	84.9 (46.3–161.6)	1.80 ± 0.05	1.28 ± 0.2
*Bt*60	Madinah	14.7 (12.5–17.5)	58.2 (38.1–89.9)	2.75 ± 0.10	0.13 ± 0.1
*Bt*63	Makkah	3.9 (3.4–4.4)^*^	15.7 (12.3–20.3)	2.70 ± 0.06	0.7 ± 0.2
*Bt*67	Madinah	6.9 (5.9–8.2)^*^	50.6 (31.5–83.4)	1.90 ± 0.05	0.6 ± 0.1
*Bt*68	Madinah	5.2 (4.5–5.8)^*^	21.6 (16.6–28.5)	2.64 ± 0.06	1.05 ± 0.3

^*^Asterisks indicate significant differences compared to the reference *Bti-*H14

^a^LC_50_ and LC_95,_ Lethal concentration that kills 50 and 95% of larval population (*n* = 20), respectively (calculated by Probit analysis and reported with 95% confidence intervals)

^b^CFU, colony forming unit (mean ± standard error). Each bioassay comprised five concentrations, one replicate for each concentration, and were repeated five times (*n* = 5)

### 16S rRNA gene analysis

A total of 19 larvicidal and 4 non-larvicidal native *Bt* isolates with varying biochemical profiles were used for 16S rRNA gene analysis, along with the reference strain *Bti*-H14 and *B. cereus* (ATCC1177) strain. Amplified target bands (~1,550 bp), were detected by agarose gel electrophoresis, for all tested *Bt* strains and *B. cereus* [24] before being sequenced. In addition, 16S rRNA genome reference sequences from *Lysinibacillus sphaericus*, *Bacillus pumilus* and *B. megatorium* were used as outgroups in a neighbour-joining analysis.

**Fig. 1:**
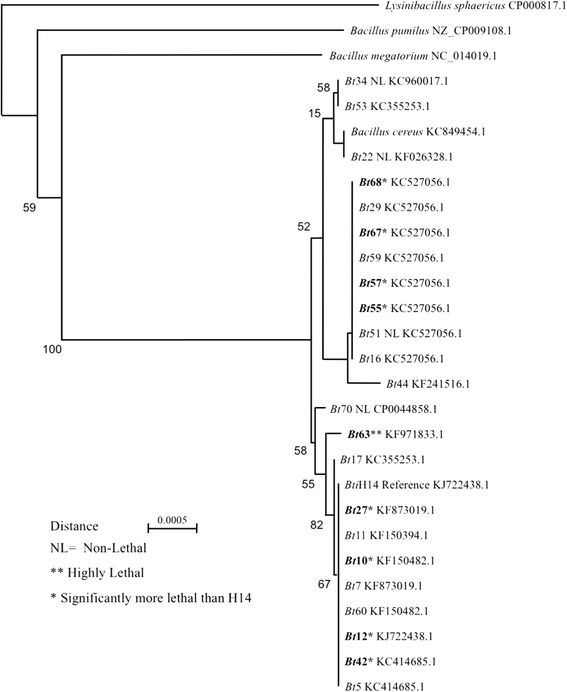
Neighbour-joining tree describing the degree of genetic similarity of native larvicidal and non-larvicidal (NL) isolated from Saudi Arabia, compared to sequences from the *Bti*-H14 and *B. cereus* reference strain. Outgroups include the GRAM-positive bacteria *Lysinibacillus sphaericus*, *Bacillus pumilus* and *B. megatorium*. Bootstrap values are indicated as well as isolates that were significantly more larvicidal (*), as well as the highly lethal *Bt*63 isolate (**)

Initial results of the BLAST analysis of the nucleotide sequence proved that the 24 tested isolates, including the colony grown *Bti-*H14 and *B. cereus* strains, were all highly homologous to the *B. thuringiensis* genome (99.5% homology in all cases). Furthermore, the neighbour-joining analysis confirmed that all new *Bt* isolates, including larvicidal and non-larvicidal ones, belonged to the previously described *B. cereus* and *B. thuringiensis* clade also referred to as *B. cereus* (*s.l*.) complex [41] and shared a high degree of genetic similarity (Fig. 1). This included several isolates significantly more toxic than the reference Bti-H14 reference. The highly toxic *Bt*63 isolate was also closely related to *Bti*-H14. In summary, all tested strains, were found to be relatively homogenous and to share a high degree of phylogenetic relatedness.

### *cry*, *cyt* and *chi* gene PCR-profiling

**Fig. 2:**
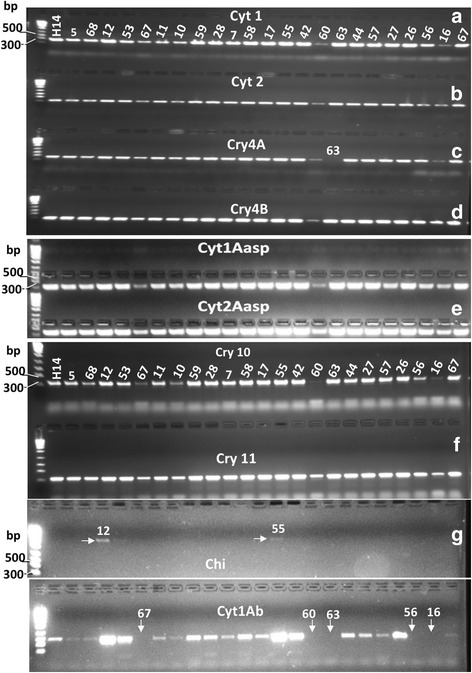
Photographs of agarose electrophoresis gels (2%) for PCR-profiling with a panel of *Cry*, *Cyt* and *Chi* gene primers. From left to right and for all panels: Lane 1: 100 bp ladder; Lane 2: reference *Bti*-H14; Lanes 3–25: the 23 native *Bt* strains indicated by their corresponding identification numbers (see Table 3). In **a**, **b**, **d**–**f**, all 23 native *Bt* strains including *Bti*-H14 displayed positive amplification of *Cyt*1, *Cyt*2, *Cry*4B, *Cry*10, *Cry*11, *Cyt*1Aa and *Cyt*2Aa. In **c**, all strains were positive for *Cry*4A except *Bt*63. In **g**, all *Bt* strains were PCR negative for *Chi* gene except *Bt*-12 and 55; whereas all *Bt* strains were PCR positive for *Cyt*1Ab gene, except the native isolates coded 67, 60, 63, 56 and 16

Each of these 23 isolates and the *Bti*-H14 strain were tested for positive amplification of the *cry* and *cyt* and *chi* genes specific to Diptera using a battery of previously-described universal and specific primers [25, 31–34] (Additional file 2: Table S2). All examined isolates yielded amplicons of the predicted size for *cry4*, *cry11*, *cyt1* and *cyt2* (Fig. 2a–f). Similarly, and except for the highly mosquitocidal *Bt*63 which showed no amplification for *cry4Aa*, most isolates yielded the expected amplicons for *cry4Aa*, *cry4Ba*, *cry10*, *cyt1Aa* and *cyt2Aa*. This was also true for the reference *Bti-*H14 strain. *Bt*-63 as well as the isolates *Bt*16, *Bt*56, *Bt*60 and *Bt*67 did not yield bands for the *cyt*1Ab gene (Fig. 2g). None of the isolates showed amplification for the *cyt2Ba*, *cry2*, *cry17* + *27*, *cry24* + *40*, *cry25*, *cry29* and *cry30* genes (data not shown). Only two native *Bt* isolates (*Bt-*12 and *Bt-*55) showed amplification of *chi* gene PCR products at the expected size (~2,027 bp) (Fig. 2g). The isolate (*Bt-*55) had the same *cry* and *cyt* genes profile as *Bti-*H14 but had significantly higher larvicidal activity against *Anopheles*, supporting the idea of a Chitinase-induced additive or synergistic larvicidal effect (Table 4). However, several native *Bt* isolates (e.g. *Bt*17, *Bt*29, *Bt*44) with significantly lower larvicidal activity than the *Bti-*H14 reference had identical *cry* and *cyt* genes profile to it and this was also true for several of the strains with significantly higher mosquitocidal activity (Table 4). With the exception of, *Bt*63, *Bt*55 and *Bt*67 strains, the remaining four strains showed *cry* and *cyt* gene amplification profiles similar to *Bti*-H14. Thus, whilst PCR-amplification profiles provided some information about possible genetic variation in the determinants of mosquitocidal activity, the large remaining variation in bioactivity combined with possible occurrence of null-alleles called for more powerful bio-molecular approaches.

**Table 4:** PCR-amplification profiles for the *cry*, *cyt* and *chi* genes of eight native *Bt* strains significantly more bioactive that the *Bti*-H14 reference strain against *An. gambiae* larvae

Strain/Accession No.^a^	Mean LC_50_ (μg/ml) (range)^b^	Positive *cry*, *cyt*, and *chi* genes							
		*cry4Aa*	*cry4Ba*	*cry10*	*cry11*	*cyt1Aa*	*cyt1Ab*	*cyt2Aa*	*chi*
*Bti-*H14 - KJ722438.1	13.33 (11.2–15.9)	+	+	+	+	+	+	+	no
*Bt*10 - KF150482.1	4.96 (4.41–5.58)^*^	+	+	+	+	+	+	+	no
*Bt*42 - KC414685.1	5.90 (5.2–6.8)^*^	+	+	+	+	+	+	+	no
*Bt*55 - KC527056.1	4.11 (3.6–4.6)^*^	+	+	+	+	+	+	+	+
*Bt*57 - KC527056.1	7.40 (6.5–8.25)^*^	+	+	+	+	+	+	+	no
*Bt*58	5.73 (5.06–6.5)^*^	+	+	+	+	+	+	+	no
*Bt*63 - KF971833.1	3.90 (3.4–4.4)^*^	no	+	+	+	+	no	+	no
*Bt*67 - KC527056.1	6.90 (5.9–8.2)^*^	+	+	+	+	+	no	+	no
*Bt*68 - KC527056.1	5.16 (4.5–5.8)^*^	+	+	+	+	+	+	+	no

*Abbreviation*: *no* indicates absence of PCR-amplification

^*^Asterisks indicate significantly greater bioactivity compared to *Bti*H14 (see Table 3)

^a^16s RNA sequences accession number when applicable

^b^Mean and range of lethal concentrations (LC_50_)

### SDS-PAGE analysis

**Fig. 3:**
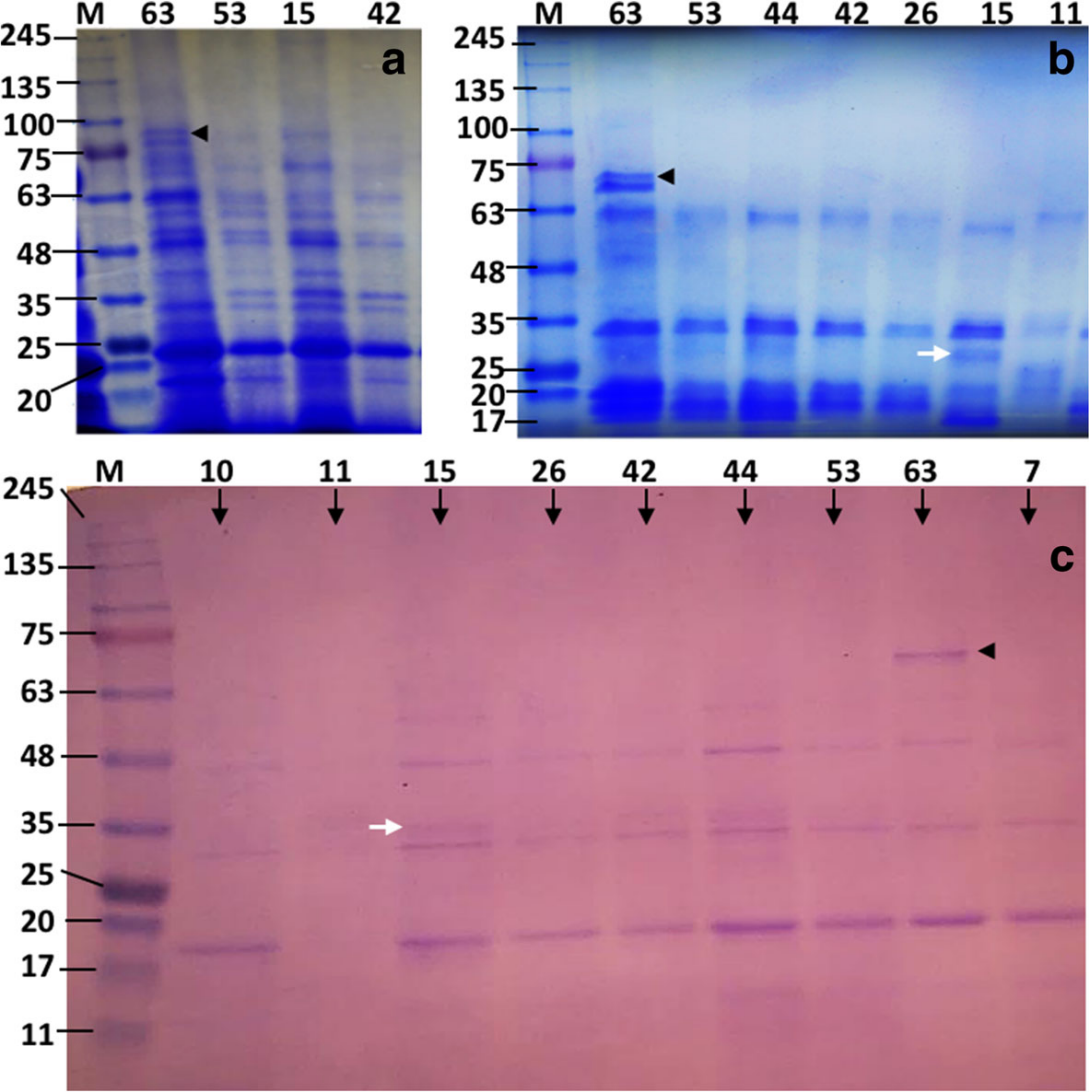
SDS-PAGE profiles of whole parasporal crystals/spores mixtures. **a** Profiles after dissolution of protein crystals at alkaline pH (10.5–11). **b** Profiles following pH-neutralization. **c** Profiles after trypsin-treatment (silver stain). The reference *Bt*-H14 is labelled as Lane 15 and represented native *Bt* isolates labelled with their respective identification numbers (see Table 4). Lanes M: protein molecular mass markers (245 to 11 kDa). Across all three conditions, SDS-PAGE profiles were distinct between the highly bio-active native *Bt*-63 isolate and reference *Bti*-H14 with *white* and *black* arrows indicating bands present in one but not the other

Further comparisons between the highly mosquitocidal *Bt*63 strain, the known reference *Bti*-H14 and other native mosquitocidal isolates were performed through SDS-PAGE analysis of the protein profiles of sporulated cultures (Fig. 3). Three experimental approaches were conducted: (i) direct spores/crystals suspension (collected 72 h after sporulation), SDS-solubilisation, centrifugation, immediately followed by SDS-PAGE analysis [42] (Fig. 3a); (ii) previously-collected spores/crystals protein solubilisation (pH 10.5–11), centrifugation to remove spores, pH-neutralization, protein determination, then SDS-PAGE analysis (Fig. 3b), and (iii) SDS-PAGE analysis with trypsin–activated solubilized protein (1 μg trypsin/20 μg protein) as mentioned above but with silver staining (Fig. 3c). Overall, the native *Bt* isolates showed similar protein-pattern as that of *Bti-*H14 in all three experimental conditions. However, the protein profile of *Bt*63 was strikingly different from that shown for the reference strain *Bti-*H14 here and in a previous report [42]. Whilst *Bt*63 shared several bands (≥ 75%) with *Bti*-H14, it had two distinct additional bands with molecular masses of 68 to 74 and 99 kDa, and was missing one band of around 28 kDa, the expected size for the missing *Cyt*1Ab, suggesting that it is a natural variant of *Bti-*H14 (Fig. 3c).

### Further characterisation of the toxic *Bt-*63 strain

**Fig. 4:**
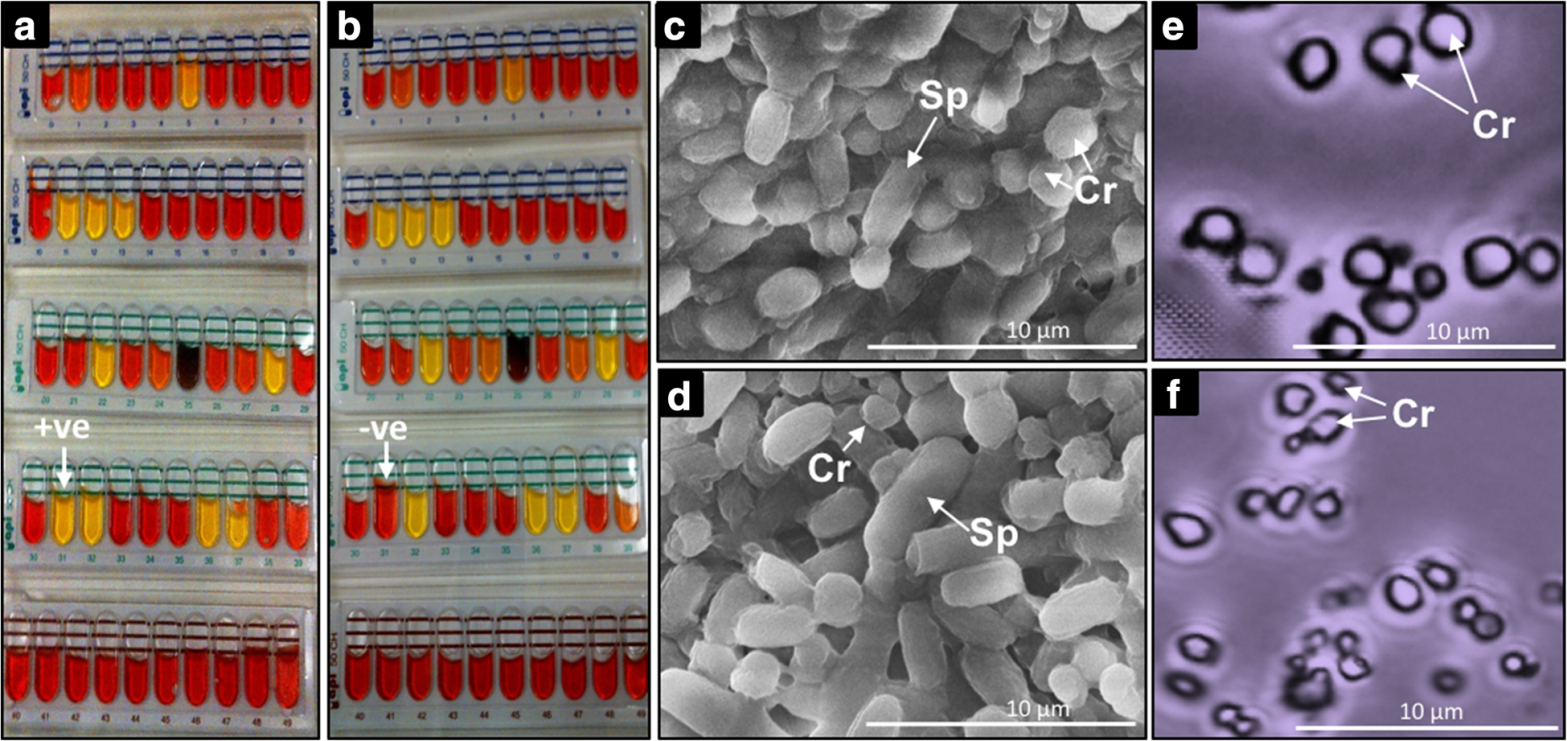
Comparisons among the native *Bt*63 and the reference strain *Bt*-H14 through biochemical profiling, scanning electron micrography and phase-contrast microscopy. In **a**, biochemical profiling with the API 50CH system shows that the *Bt*63 isolate produces acid from sucrose (indicated by *arrow*), whereas in **b** *Bti*-H14 is negative (*arrow*); all other 49 biochemical reactions were similar. In **c** and **d**, scanning electron micrograph (×10,000) of *Bt*63 reveals its larger *Cry* crystals (Cr) and smaller spores (Sp) than those *Bti-*H14. In **e** and **f**, the phase-contrast micrographs of sucrose gradient-separated *Cry* Crystals (Cr) from *Bt*63 appear, comparatively, larger than those of *Bti-*H14. *Scale-bars*: **c**, **d**, 1 μm; **e**, **f**, 10 μm

Biochemical typing of both strains using the in API 50CH System revealed that the only notable difference between *Bt*63 and the reference *Bti*-H14 was that the former produces acid from sucrose whereas the *Bt*-H14 does not (Fig. 4a-b).

Detailed morphometric comparisons of largest spherical to ovoid crystals and spores (5 field readings for each) collected from growth on NYSM liquid medium from the highly larvicidal *Bt*63 and *Bti*-H14 reference were conducted by Scanning Electron Microscopy (SEM) [24] (Fig. 4c–d). The average size of spores (Length ± SD × Width ± SD) tended to be larger in *Bti*-H14 (1.75 ± 0.12 × 0.964 ± 0.049 μm) than in *Bt*63 (1.60 ± 0.15 × 0.931 ± 0.076 μm). Comparing the smallest crystals (5 field readings for each) found in both strains showed that these reached a smaller size in *Bti*-H14 (0.618 ± 0.043 × 0.510 ± 0.096 μm) than in *Bt*63 (0.702 ± 0.047 × 0.621 ± 0.065 μm). Large crystals were also comparatively smaller in *Bti*-H14 (0.911 ± 0.080 × 0.792 ± 0.082 μm) than in *Bt*63 (1.008 ± 0.162 × 0.901 ± 0.125 μm). These differences were also evident from phase contrast microscopy of sucrose gradient crystal separation (Fig. 4e-f) [43].

## Discussion

This study was carried out to create a collection of native *Bt* isolates that can potentially be used for developing bio-control tools to help fight mosquito-borne diseases in Saudi Arabia. Under the assumption that sampling from different countries and region might uncover novel genetic diversity and toxic potential, *Bt* isolates were obtained from a variety of samples collected in different ecosystems in 16 regions of Saudi Arabia as they have extensive irrigation systems which creates an abundance of suitable mosquito breeding sites.

A total of 68 *Bt* isolates were successfully isolated from 300 samples, which often contained more than one *Bt* isolates from the same sample. Most of these isolates (47.27%) originated from soil samples collected from old urban regions, such as those from Al-Madinah and Makah. This contrasted with *Bt* negative samples, which came from less populated new city regions (mostly surrounded by vast desert land). About 35.3% of recovered isolates were shown to have mosquito larvicidal activity and almost half of these came from Al-Madinah region, the oldest urban area in Saudi Arabia. This could be attributed to the extensive irrigation systems in that region which creates an abundance of suitable mosquito breeding sites. However, the presence of *Bt* is not strictly associated with the presence of mosquitoes and other insect in the soil, as past studies have found it in soils with little or no insect activity, and sometimes could not find it from soils with high insect activity [7]. Furthermore, and considering the fact that commercially available *Bti* formulations do not last in the environment for long periods of time and that they have only been very rarely used in field evaluation in Saudi Arabia [42, 44, 45], the 68 *Bt* isolates can be considered as part of the indigenous microflora of the areas explored.

As observed in other surveys [4, 25, 40], a large amount of variation in crystal morphology was observed among the 68 *Bt* field isolates. Furthermore, and in agreement with Martin et al. [40], the presence of anamorphous and/or irregular crystals in 23 (32%) of our isolates correlated with high mosquito larvicidal activity. In their study of the biochemical activity profiles of 3,639 *Bt* isolates from different countries, the same authors [40] suggested that urease producers were strongly associated with bipyramidal crystals toxic to Lepidoptera, whereas isolates with amorphous and/or irregular crystals tended to be toxic to Diptera, with general low metabolic activity, positive for acid production from starch and lecithinase and/or esculine hydrolysis [40].

In addition to their crystal shape, the 23 native larvicidal *Bt* strains showed similarities in their biochemical activity. Thus, all of these 23 strains were hemolytic for sheep RBCs, actively motile, positive for esculin hydrolysis, gelatinase, or phospho-lipase (lecithinase) activity. These strains, however, failed to produce acid from salicin and sucrose, except two strains (coded *Bt*-7 and *Bt*-63) which were sucrose positive. This is again in agreement with Martin et al*.* [40] who found that *Bt*i-H14-like strains were positive for starch and lecithinase but negative for salicin and sucrose. Despite similarities in the crystal shape and biochemical profiles of larvicidal isolates, detailed testing of their larvicidal activity revealed much variation in their toxicity to the malaria mosquito *An. gambiae*. Thus, out of the 23 *Bt* isolates, 8 displayed significantly higher toxicity against *An. gambiae* than the reference *Bti*-H14 strain, whilst the remaining 15 isolates did not significantly differ from *Bti*-H14 in their larvicidal bioactivity, despite varying in their spore counts.

Amongst the *Bt* isolates with significantly enhanced larvicidal activity identified in this study, isolate *Bt*63 was the most toxic with a bioactivity against *An. gambiae* larvae 3.4-fold higher than H14. This isolate showed similar crystal shapes to those of *Bti*-H14, which included small spherical, triangular, merged ovoid double spherical and/or small double merged triangular crystals. However, SEM-morphometric measurements of the largest ovoid spherical crystals and of spores revealed remarkable differences between the two *Bt* strains, the former being larger in *Bt*63 and the later larger in *Bti*-H14.

In a survey conducted in Japan, Saitoh et al. [46] screened 1,449 local *Bt* isolates for larvicidal activity against the mosquito *Anopheles stephensi* and found the majority of their isolates (97.2%) exhibited little or no larvicidal activity. In addition, the 2 most active Japanese isolates were 13-fold and 23-fold less active than the international reference *Bti*-H14. Nowadays, several *B. thuringiensis* strains with good mosquitocidal activity have been isolated from various parts of the world such as *Bt* subsp. *morrisoni* PG-14, *jegathesan*, *kyushuensis*, *medellin*, *darmstadiensis* 73-E10-2 or *Bt* S2160. In all these isolates, a cytolytic toxin, generally 25 to 30 kDa, was also produced in addition to different Cry endotoxins; yet, except for strain PG-14, they were all less toxic to mosquitoes than *Bti*-H14.

In Latin America, Ibarra et al. [31] isolated 4 *Bti*-like strains with higher mosquitocidal toxicity than *Bti*-H14 despite sharing similar PCR profiles for the *cry* and *cyt* gene-toxins. The authors pointed out that this higher bioactivity could be due to: (i) the *cry* and *cyt* genes detected by PCR may be encoding novel protein variants; (ii) the *cry* and *cyt* genes may be identical, but their expression levels may be different; or (iii) an undetected factor or protein may be responsible for their higher activity. The latter explanations may hold true for the native *Bt*55 strain described in this study, which had enhanced mosquitocidal activity, a similar *cry* and *cyt* gene profile as that of *Bti*-H14, but also possessed the *Chi* gene. The chitinase gene has been shown to elevate the mosquitocidal effect by perforating the peritrophic membrane, thereby increasing the accessibility of the Cry and Cyt toxins to receptors on the epithelial membrane [7, 47]. Abdullah et al. [48] further experimentally demonstrated that transferring the chitinase gene from *B. subtilis* to a *Bt* strain improved its insecticidal bioactivity.

At the physiological and molecular levels, the highly toxic native *Bt*63, differed from *Bti*-H14 by having larger hemolytic and lecithinase activities and utilizing sucrose, which opens the possibility of bio-insecticide production using fermentation media such as locally-produced date molasses. In the 16S rRNA neighbour-joining tree *Bt*63 was more closely related to *Bti*-H14 than to *B. cereus*, albeit not as close as some of the other isolates characterized in this study, which may also suggest a recent independent evolutionary trajectory. In addition, the SDS-PAGE profiles obtained from different total protein extractions from crystal/spore mixture, were all strikingly different from those of *Bti*-H14 despite sharing many protein bands in Cry and Cyt regions [43]. In all cases, *Bt*63 showed an extra band of (68–72 to 99 kDa), possibly belonging to the naturally truncated Cry11, truncated fragment of a second *cry*4B gene or of *cry*60 genes [49].

PCR-detection profiles of *cry* and *cyt* genes also revealed that *Bt*63 shared the *cry4Ba*, *cry10*, *cry11A*, *cyt1Aa* and *cyt2Aa* bands with *Bti*-H14 but not those for the *cry4Aa* and *cyt1Ab* gene. Our results also suggest that the amount of crystals/spore was higher in the native *Bt*63 than in *Bti*-H14. The SEM observations of larger crystals and smaller spores combined with enhanced larvicidal activity (3.4-fold) but lower overall spore count (6-fold) in *Bt*63 compared to *Bti*-H14 suggest that the loss of the large *cry4Aa* could have been adaptively compensated by increased production and crystallization of *cry* and *cyt* toxins in the former strain. However, given the complexity and variety of transcriptional, post-transcriptional and post-translational processes involved in the synthesis of δ-endotoxins and sporulation process [2], this hypothesis remains to be further investigated. We believe that the native *Bt*63 strain could harbour the best combination of *cry* and *cyt* genes, resulting in elevated synergistic effects and, possibly, minimizing Cry competition for less specific receptors [18] or Cyt competitions for non-specific larval gut’s receptors, which are key to synergetic oligomer pore-formation. In support for this hypothesis, Correa et al. [50] found that the combination of activated Cyt2Ba and Cry11A showed higher toxic activity than the two toxins activated solely, or a combination of Cry4Aa, Cry11A and Cyt2Ba used concomitantly, suggesting Cry4Aa competition and/or antagonistic effect. Therefore, in this study, the natural loss of Cry4Aa in the native *Bt*63 could increase the synergistic effect of Cry11Aa and Cyt1Aa and/or Cyt2Aa due to absence of Cry4Aa competition.

Overall, the data suggest that the native *Bt*-63 strain is an environmental variant closely-related to *Bti*-H14, but which has lost the capacity to produce Cry4Aa and Cyt1Ab toxins, resulting in increased toxicity against dipteran larvae. Further studies are needed to test that hypothesis such as, for example, plasmid profile detection, plasmid and/or isolation, and/or cloning and transfer to an acrystalliferous *Bt* strain, and testing of mosquito toxicity of the resulting strain. Such studies might reveal the acquisition by *Bt*-63 of other *cry* and/or *cyt* genes that could help explain the distinct SDS-PAGE profiles obtained.

In agreement with the hypothesis that the loss of a Cry toxin can sometimes translate in increased larvicidal activity, Ibarra et al. [31] found that a native *Bt* strain (LBIT348) from Mexico which lacked Cry10 compared to a reference *Bt-israelensis* strain exhibited significantly higher mosquitocidal activity than the later strain*.* That is, despite the fact that the combination of Cry4, Cry10, Cry11 and Cyt toxins, is known to be very potent against mosquitoes [31]. Here, *Bt*63 contained the *cry4Ba*, *cry10*, *cry11A*, *cyt1Aa* and *cyt2Aa* genes, but lacked *cry4A*. In Iran, Jouzani et al. [32] isolated 128 *Bt* strains from different ecological regions of Iran with dipteran-specific toxicity and detected *cry4B* in 60% of isolates and *cry4C* or *cry4D* in 40% of isolates, whereas *cry4A* was not detected in any strains.

## Conclusions

Various studies have emphasized various synergetic interactions between different combinations of Cry and Cyt proteins and how these can vary in their effectiveness against different mosquito taxa [51–54]. We believe that the discovery of the *Bt*-63 strain, native to the oldest city of Makkah in Saudi Arabia and isolated from its sewage water, may prove crucial for future bio-insecticide production against mosquito-vectors. This is due to its greatly enhanced mosquitocidal activity against *An. gambiae,* combined with possible economic and environmental advantages thanks to its, comparatively, larger Cry and Cyt toxin crystals, and its capacity to metabolize sucrose which opens the possibility to grow it on cheap or wastes-based fermentation media.

## Additional files

Additional file 1: Table S1. GenBank accession numbers for the 16S rRNA gene sequences of *Bt* strains and isolates and Gram-positive outgroup species of bacteria used in the neighbour-joining cluster analysis (DOCX 13 kb)
Additional file 2: Table S2. Universal (UN) and specific (sp) primers, amplicon size (bp), and annealing temperature T_*a*_ used for PCR-profiling of the *cry*, *cyt* and *chi* genes. (DOCX 16 kb)
