# Supplementary material for: Isolation and characterization of native Bacillus thuringiensis strains from Saudi Arabia with enhanced larvicidal toxicity against the mosquito vector Anopheles gambiae (s.l.)
Source: Parasit Vectors. 2016 Dec 19;9:647. doi: 10.1186/s13071-016-1922-6 (PMC5168711; doi:10.1186/s13071-016-1922-6)
Supplement: Additional file 1: Table S1. — GenBank accession numbers for the 16S rRNA gene sequences of Bt strains and isolates and Gram-positive outgroup species of bacteria used in the neighbour-joining cluster analysis (DOCX 13 kb) [file 13071_2016_1922_MOESM1_ESM.docx]

**Additional file 1: Table S1.** GenBank accession numbers for the 16S rRNA gene sequences of *Bt* strains and isolates and Gram-positive outgroup bacteria species used in the neighbour-joining cluster analysis

| **Specie/Strain/Isolate** | **Accession number** |
| --- | --- |
| *Bacillus megaterium* QM strain B1551 | NC_014019.1 |
| *Bacillus pumilus* strain GR-8 | NZ_CP009108.1 |
| *Lysinibacillus sphaericusc* strain C3-41 | CP000817.1 |
| *Bacillus cereus* | KC849454.1 |
| *Bacillus thuringiensis israelensis* H14 reference strain | KJ722438.1 |
| *Bt*5 isolate | KC414685.1 |
| *Bt*7 isolate | KF873019.1 |
| *Bti*10 isolate | KF150482.1 |
| *Bt*11 isolate | KF150394.1 |
| *Bt*12 isolate | KJ722438.1^a^ |
| *Bt*16 | KC527056.1 |
| *Bt*17 isolate | KC355253.1 |
| *Bt*22 | KF026328.1 |
| *Bt*27 isolate | KF873019.1 |
| *Bt*29 | KC527056.1 |
| *Bt*34 | KC960017.1 |
| *Bt*42 isolate | KC414685.1 |
| *Bt*44 | KF241516.1 |
| *Bt*60 isolate | KF150482.1 |
| *Bti*63 | KF971833.1 |
| *Bt*68 | KC527056.1 |
| *Bt*70 | CP0044858.1 |

^a^Isolate with 16S rRNA sequence identical to H14
