# Supplementary material for: Isolation and characterization of native Bacillus thuringiensis strains from Saudi Arabia with enhanced larvicidal toxicity against the mosquito vector Anopheles gambiae (s.l.)
Source: Parasit Vectors. 2016 Dec 19;9:647. doi: 10.1186/s13071-016-1922-6 (PMC5168711; doi:10.1186/s13071-016-1922-6)
Supplement: Additional file 2: Table S2. — Universal (UN) and specific (sp) primers, amplicon size (bp), and annealing temperature Ta used for PCR-profiling of the cry, cyt and chi genes. (DOCX 16 kb) [file 13071_2016_1922_MOESM2_ESM.docx]

**Additional file 2: Table S2.** Universal (UN) and specific (sp) primers, amplicon size (bp), and annealing temperature T*_a_* used for PCR-profiling of the *cry*, *cyt* and *chi* genes

| **Primer** | **Sequence 5'-3'** | **Gene** | **Size (bp)** | **T*_a_* (°C)** | | **Reference** |
| --- | --- | --- | --- | --- | --- | --- |
| *cry2* (UN) | F: GAGTTTAATCGACAAGTAGATAATTT  R: GGAAAAGAGAATATAAAAATGGCCAG | *cry2Aa* | 526 | 50 | [31] | |
|  |  | *cry2Ab* | 526 |  |  |  |
|  |  | *cry2Ac* | 520 |  |  |  |
|  |  | *cry2Ad* | 500 |  |  |  |
| *cry4* (UN) | F: GCATATGATGTAGCGAAACAAGCC  R: GCGTGACATACCCATTTCCAGGTCC | *cry 4A2* | 439 | 58 | [32] | |
|  |  | *cry 4B4* | 439 |  |  |  |
| *cry4A* (sp) | F: TCAAAGATCATTTCAAAATTACATG  R: CGGCTTGATCTATGTCATAATCTGT | *cry 4Aa* | 459 | 50 | [32] | |
| *cry4B* (sp) | F: CGTTTTCAAGACCTAATAATATAATACC  R: CGGCTTGATCTATGTCATAATCTGT | *cry4Ba* | 321 | 50 | [32] | |
| *cry10* (sp) | F: TCAATGCTCCATCCAATG  R: CTTGTATAGGCCTTCCTCCG | *cry 10* | 348 | 51 | [32] | |
| *cry11* (UN) | F: CGCTTACAGGATGGATAGG  R: GCTGAAACGGCACGAATATAATA | *cry11Aa* | 342 | 50 | [31] | |
|  |  | *cry11Ba* | 342 |  |  |  |
|  |  | *cry11Bb* | 452 |  |  |  |
| *cyt1* (UN) | F: CCTCAATCAACAGCAAGGGTTATT  R: TGCAAACAGGACATTGTATGTGTAATT | *cyt1Aa* | 477 | 52 | [31] | |
|  |  | *cyt1Ab* | 480 |  |  |  |
|  |  | *cyt1Ba* | 477 |  |  |  |
| *cyt2* (UN) | F: ATTACAAATTGCAAATGGTATTCC  R: TTTCAACATCCACAGTAATTTCAAATGC | *cyt2Aa* | 356 | 50 | [31] | |
|  |  | *cyt2Ba* | 355 |  |  |  |
|  |  | *cyt2Bb* | 355 |  |  |  |
|  |  | *cyt2Ca* | 355 |  |  |  |
| *cry17*+*27* | F: CATTGTTCTACTTGGTATAA  R: GATACAATTACATCTCCTCCTGTA | *cry17Aa* | 832 | 47 | [33] | |
|  |  | *cry27Aa* | 895 |  |  |  |
| *cry24*+*40* | F: TTATCAATGTTAAGGGATGC  R: ACTGGATCTGTGTATATTTTCCTAG | *cry24Aa* | 304 | 48 |  |  |
|  |  | *cry40Aa* | 366 |  |  |  |
| *cry25* (sp) | F: GGCTTCTAGATCAGGAGATGG  R: CATCATAATCAGAGCGCAGG | *cry25* | 560 | 53 |  |  |
| *cry29* (sp) | F: TCAGCTCCAATAACTGGTG  R: GCATGTCATCCCCTTGTCTA | *cry29* | 451 | 50 |  |  |
| *cry30* (sp) | F: AACTCACACATCCTCCATCG  R: ATCGGAAGGCAATCATTCG | *cry30* | 265 | 50 |  |  |
| *cyt2Ba* (sp) | F: GGATCCATGCACCTTAATTTGAATAATTTT  R: GGATCCTTAGTGGTGGTGGTGGTGGTGATACGATTTTATTGGAT | *cyt2Ba* | 800 | 52 |  |  |
| *cyt1Aa* (sp) | F: AACTCAAACGAATAACCAAG  R: TGTTCCTTTACTGCTGATAC | *cyt1Aa* | 300 | 53 |  |  |
| *cyt1Ab* (sp) | F: AAGCAAGGGTTATTACATTACG  R: CCAATACTAAGATCAGAGGG | *cyt1Ab* | 698 | 54 |  |  |
| *cyt2Aa* (sp) | F: GCATTAGGAAGACCATTTG  R: AAGGCTAAGAGTTGATATCG | *cyt2Aa* | 361 | 53 |  |  |
| *chi* | F: ATGGTCATGAGGTCTC  R: CTATTTCGCTAATGACG | *chi* | 2027 | 45 |  |  |
